# Functional Relationship between Skull Form and Feeding Mechanics in *Sphenodon*, and Implications for Diapsid Skull Development

**DOI:** 10.1371/journal.pone.0029804

**Published:** 2011-12-28

**Authors:** Neil Curtis, Marc E. H. Jones, Junfen Shi, Paul O'Higgins, Susan E. Evans, Michael J. Fagan

**Affiliations:** 1 Medical and Biological Engineering Research Group, Department of Engineering, University of Hull, Hull, United Kingdom; 2 Research Department of Cell and Developmental Biology, University College London, London, United Kingdom; 3 Hull-York Medical School, University of York, York, United Kingdom; Raymond M. Alf Museum of Paleontology, United States of America

## Abstract

The vertebrate skull evolved to protect the brain and sense organs, but with the appearance of jaws and associated forces there was a remarkable structural diversification. This suggests that the evolution of skull form may be linked to these forces, but an important area of debate is whether bone in the skull is minimised with respect to these forces, or whether skulls are mechanically “over-designed” and constrained by phylogeny and development. Mechanical analysis of diapsid reptile skulls could shed light on this longstanding debate. Compared to those of mammals, the skulls of many extant and extinct diapsids comprise an open framework of fenestrae (window-like openings) separated by bony struts (e.g., lizards, tuatara, dinosaurs and crocodiles), a cranial form thought to be strongly linked to feeding forces. We investigated this link by utilising the powerful engineering approach of multibody dynamics analysis to predict the physiological forces acting on the skull of the diapsid reptile *Sphenodon*. We then ran a series of structural finite element analyses to assess the correlation between bone strain and skull form. With comprehensive loading we found that the distribution of peak von Mises strains was particularly uniform throughout the skull, although specific regions were dominated by tensile strains while others were dominated by compressive strains. Our analyses suggest that the frame-like skulls of diapsid reptiles are probably optimally formed (mechanically ideal: sufficient strength with the minimal amount of bone) with respect to functional forces; they are efficient in terms of having minimal bone volume, minimal weight, and also minimal energy demands in maintenance.

## Introduction

There is a longstanding debate as to whether bone in the skull is minimised in relation physiological loading [1], [2], or whether skulls are ‘over-designed’ and constrained by phylogeny, development, and the need to accommodate functions in addition to normal loading [3]–[5]. The skull provides a structure for jaw and neck muscle attachment and should be rigid enough to withstand the forces these muscles apply, along with accompanying feeding and other forces [6]–[8]. Exactly how the skull responds to these forces in tandem with accommodating the brain and sense organs is not fully understood. Adaptation to loads consistent with Wolff's law [9] would result in minimisation of bony material with respect to functional loading, and following a long held theory [10] the term *bone functional adaptation* [11]–[13] is often used to describe the mechanism by which bone is modelled and remodelled. Briefly, it is proposed that bone strain is the stimulus for bone modelling/remodelling [14], [15], and there is an *equilibrium window* of strain, above which bone is deposited and below which bone is removed [16]–[18]. The rules regulating bone adaptation and the exact levels at which bone is remodelled are however likely more complex, being dependent on more than just pure strain magnitudes. Strain rate, load history, bone age, disease, initial bone shape, bone developmental history, hormonal environment, diet, and genetic factors have all been highlighted as potential factors that could impact bone form [15]–[25].

**Figure 1:**
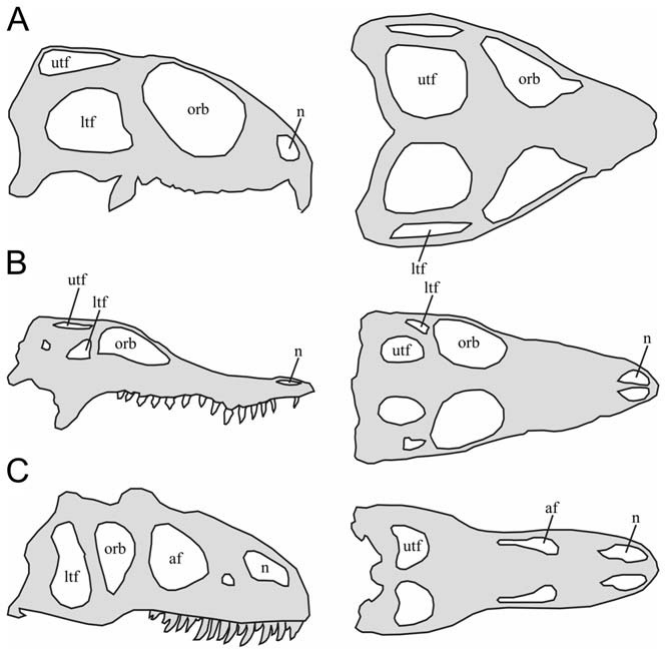
The diapsid skull form. Simplified schematic lateral and dorsal skull views of **A.** *Sphenodon* (redrawn [87]), **B.** *Crocodylus siamensis* (original drawing), **C.** *Allosaurus fragilis* (redrawn [92]). All skulls are scaled to the same length. af – antorbital fenestra; ltf – lower temporal fenestra; n – nasal opening; orb – orbital opening; utf – upper temporal fenestra.

The skull of *Sphenodon*, a New Zealand reptile, is not dominated by a large vaulted braincase like mammals, but instead comprises an open arrangement of fenestrae (windows or openings) and bony rods or struts [26], [27]. Without the constraint of a large brain and associated forces [28]–[31], the dominant loads applied to the frame-like skull of *Sphenodon* are most likely linked to feeding (i.e. muscle forces, bite forces, and jaw joint forces). This is probably also true for other diapsids that lack large brains, such as lizards, crocodiles, and theropod dinosaurs, which share comparable skull morphologies (Figure 1). Without the effect of neurocranial expansion, these frame-like skulls may be useful for investigating the correlation between skull form and bone strain under loading. Some insight into this relationship would provide new perspectives towards understanding skull form in other amniotes.

**Figure 2:**
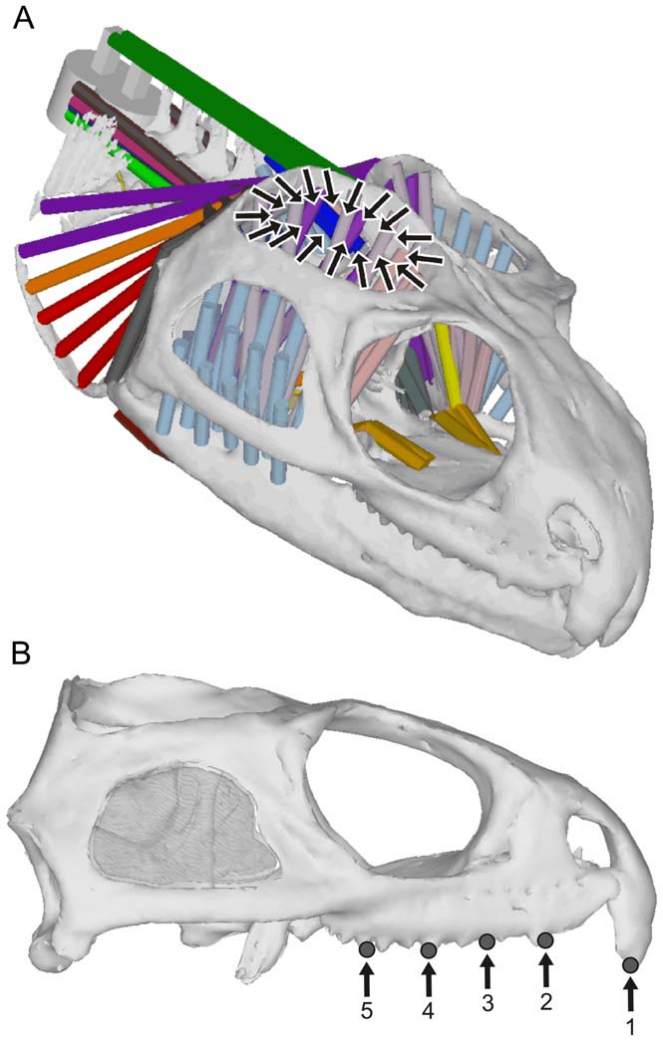
MDA model. **A.** Multibody computer model used to calculate the muscle, joint and biting forces for a series of biting simulations. Black arrows represent the location and direction of the fascial force vectors applied to the finite element model over one temporal opening. **B.** Bite locations. Bilateral (biting on both sides simultaneously) and unilateral biting (biting on one side only) at locations 2–5; bilateral biting only at location 1; ripping bites at location 2 only. Skull measures approximately 68 mm long from the tip of the premaxilla to the posterior end of the quadrate condyles.

Finite element analysis (FEA) is a virtual technique that is used to predict how a structure will deform when forces and constraints are applied to it, and has been used previously to predict stress and strain distribution within skulls [4], [27]–[29], [31]–[33], [35], [36]. However, such studies tend to apply limited loading data and are used to investigate particular aspects of skull morphology or the impact of single bites. To fully evaluate skull form it is important to take into account several different load cases, because skull form is most likely to be related to the range of physiological loads experienced by an animal rather than a single load case. We investigated the relationship between skull form and bone strain in *Sphenodon* by carrying out a series of static finite element analyses (FEAs), applying bite forces at several different bite positions. We combine the powerful computational techniques of multibody dynamics analysis (MDA) [32]–[34] and FEA, to first predict the forces acting on the skull of *Sphenodon*, and in turn analyse the strains within the skull under these forces. This enables us to evaluate the degree of correlation between skull form and three strain modes: tensile (also known as maximum and 1^st^ principal), compressive (also know as minimum and 3^rd^ principal) and von Mises (also known as equivalent and mean). Multibody dynamics analysis has recently been applied to study skull biomechanics [32]–[38], and was used here to predict muscle forces, joint forces, and bite forces in *Sphenodon* during fifteen separate biting simulations. These simulations covered a range of biting types and locations. They include four bilateral and eight unilateral bites at different tooth positions, a bite on the anterior-most chisel-like teeth, and two ripping bites that incorporate neck muscles (MDA model shown in Figure 2 and a summary of all biting simulations is given in Table 1). A corresponding set of fifteen separate FEAs was carried out to investigate the total mechanical performance of the skull under these predicted forces. Each separate FEA applied a peak static bite force and corresponding muscle and joint forces.

**Table 1:** The 15 load cases simulated during the MDA and applied in the FEA.

Load case	Type of bite	Side of skull	Bite location	Bite Location
1	unilateral	right	anterior	2
2	unilateral	right	middle	3
3	unilateral	right	posterior	4
4	unilateral	right	posterior-most	5
5	unilateral	left	anterior	2
6	unilateral	left	middle	3
7	unilateral	left	posterior	4
8	unilateral	left	posterior-most	5
9	bilateral	both	anterior	2
10	bilateral	both	middle	3
11	bilateral	both	posterior	4
12	bilateral	both	posterior-most	5
13	bilateral	both	chisel-like tooth	1
14	neck ripping bite (left)	both	anterior	2
15	neck ripping bite (right)	both	anterior	2

See Figure 2 for explanation of bite locations.

## Results

### MDA

Total bite and quadrate-articular joint forces (i.e. working and balancing sides combined) are similar whether the animal is biting unilaterally or bilaterally. However, the bite force on each side of the skull during bilateral biting is half that of unilateral biting (i.e. the total bite force is shared over both sides of the skull). Also, forces located at the balancing side joint during unilateral biting are always in excess of those at working side joint (Table 2). Bite force at the most posterior bite location (location 5 – Figure 2B) is almost 80% greater than on the chisel-like teeth at the front of the skull (location 1), whereas during unilateral biting the balancing side joint force is approximately 50% greater than the working side joint force at the most posterior bite location (location 5). Total muscle forces applied during the MDA are presented in Table 3.

**Table 2:** Bite forces and jaw joint forces predicted by the MDA.

Bite Type	Bite Location	Bite Force (N)	Working Joint Force (N)	Balancing Joint Force (N)
bilateral	1	121	540	-
bilateral	2	150	524	-
bilateral	3	165	510	-
bilateral	4	185	490	-
bilateral	5	214	462	-
unilateral	2	150	249	276
unilateral	3	166	232	276
unilateral	4	187	212	277
unilateral	5	216	183	278

Total forces are shown for bilateral bites, therefore the force on each side of the skull is approximately half that presented. Working refers to the force on the same side as the bite occurs, while balancing refers to the opposite side to which biting occurs. See Figure 2 for explanation of bite locations.

**Table 3:** Total muscle forces applied to each side of the skull during the MDA.

Muscle	Total Muscle Force (N)
Depressors (defined as 2 groups)	40
Adductors (defined as 14 groups)	448
Neck (defined as 11 groups)	158

**Figure 3:**
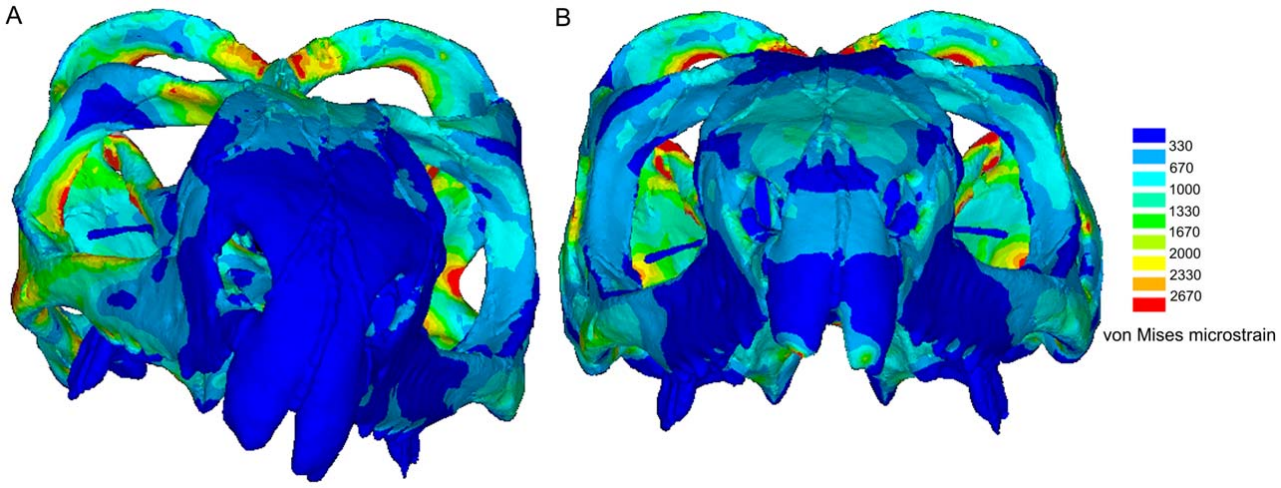
von Mises FEA plots during two single bites. Deformation and von Mises strain plots of the skull of *Sphenodon* during **A.** right unilateral biting and **B.** during bilateral biting on the anterior-most chisel-like teeth; (note the displacements are scaled by a factor of 50).

The depressor muscles were represented by two muscle groups, the adductor muscles were represented by fourteen muscle groups, and the neck muscles were represented by eleven muscle groups. This arrangement of muscles accurately depicts the anatomy of *Sphenodon*. Muscle sections are visually presented in Figure 3A, while detailed descriptions of all muscle groups are published elsewhere [18], [39].

### FEA

**Figure 4:**
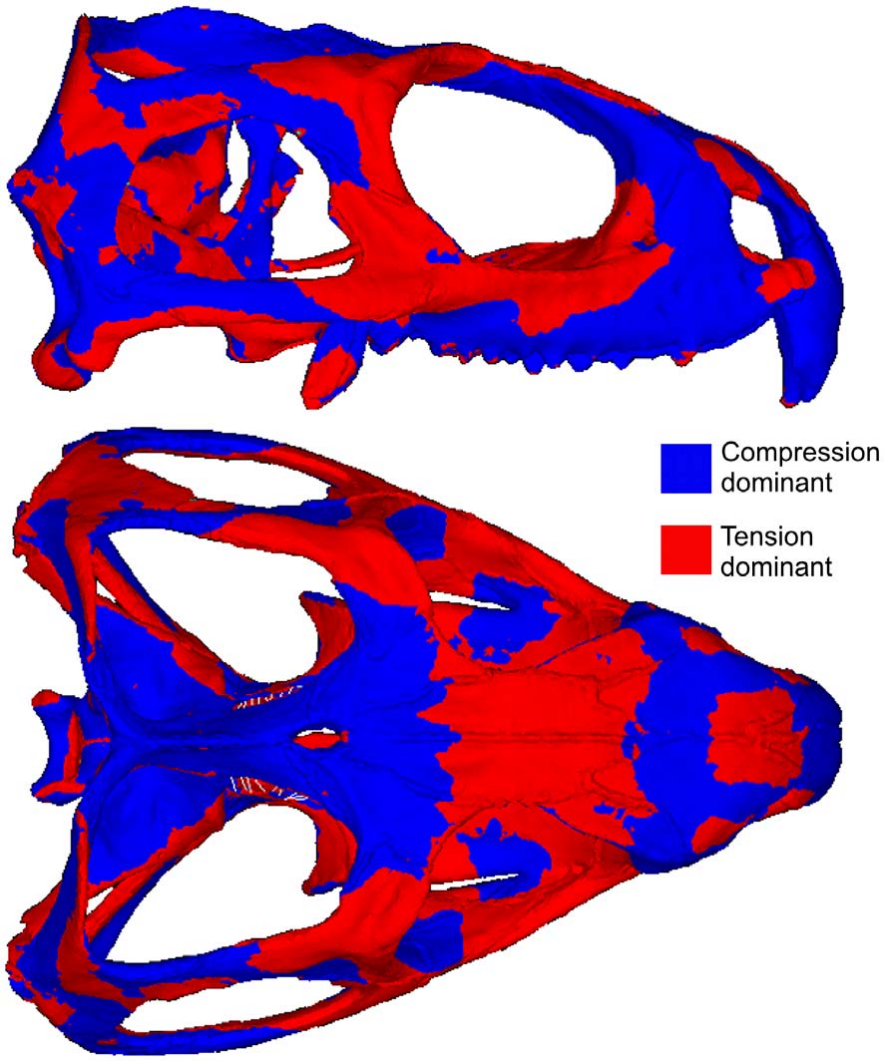
Plot of dominant strain regions. Cumulative map of peak dominant strains over all bites. Red represents regions of the skull where tensile strains are in excess of compressive strains (i.e. tensile strains are dominant), and blue represents regions where compressive strains are in excess of tensile strains (i.e. compressive strains are dominant).

**Figure 5:**
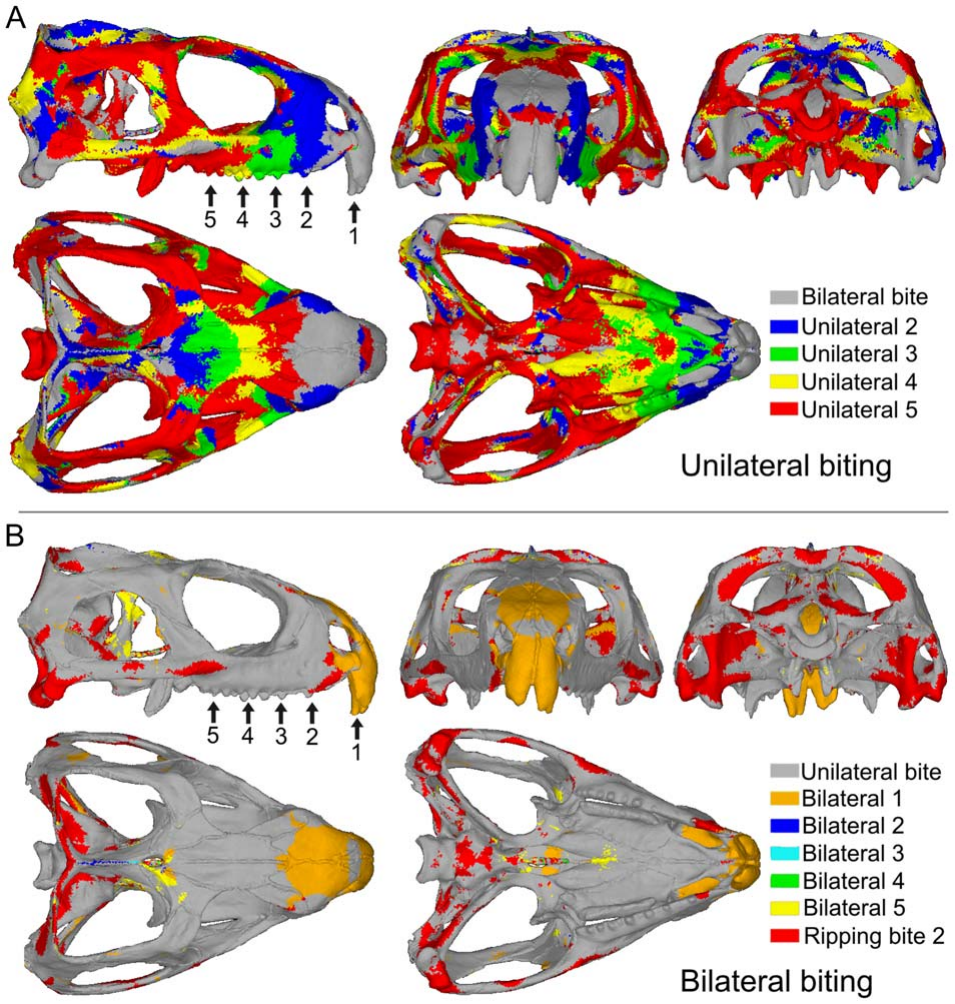
Models showing which bite location generated the highest strains in particular areas of the skull. Results based on von Mises strains. **A.** Unilateral bites and **B.** bilateral bites. (For example, in **A.** unilateral biting at location 2 was responsible for the highest strains in those areas coloured blue).

**Figure 6:**
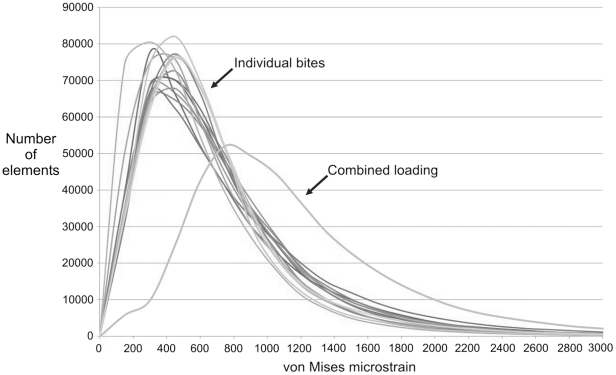
von Mises element strain distribution plots. Plot represents the number of elements within the finite element model that experience a specific strain magnitude. The plot shows the element strains from all fifteen biting simulations (labelled individual bites) and the combined loading model.

Bite location has a considerable effect on the way the skull deforms. During individual bites, strain gradients (or heterogeneous strain magnitudes) are apparent over the skull, with some regions subject to high strains and others subject to low strains (example von Mises strain plots are presented in Figure 3). As the skull deforms it experiences both compressive and tensile strains (dominant strains over all bites at specific skull locations is presented in Figure 4), and during unilateral biting these strains tend to reach their peak magnitudes (Figure 5A). In addition to the peak strains generated during unilateral bites, high strain also occurs in the nasal bone when biting on the large anterior-most chisel-like teeth, a distinctive feature of *Sphenodon* ([39]; Figure 5B, bilateral location 1). Ripping bites in which the neck muscles are highly active also strain the posterior aspects of the skull and braincase more than non-ripping bites (Figure 5B, ripping location 2). Across all simulations unilateral bites account for approximately 79% of the peak strains generated across the skull, with the posterior-most unilateral bite accounting for 60% of peak strains. Biting on the anterior-most chisel-like teeth generates approximately 9% of the peak strains in the skull, while the ripping bites were attributable for 10%. Bilateral bites (excluding biting on the anterior-most teeth) accounted for less than 2% of peak strains across the skull when all biting simulations were assessed. Strains vary over the skull at any one bite location (including those yielding the highest strains), with approximately 30% of the skull at low levels of strain below 200 microstrain, and 65% of the skull at strains of below 500 microstrain during separate bites (Figure 6).

**Figure 7:**
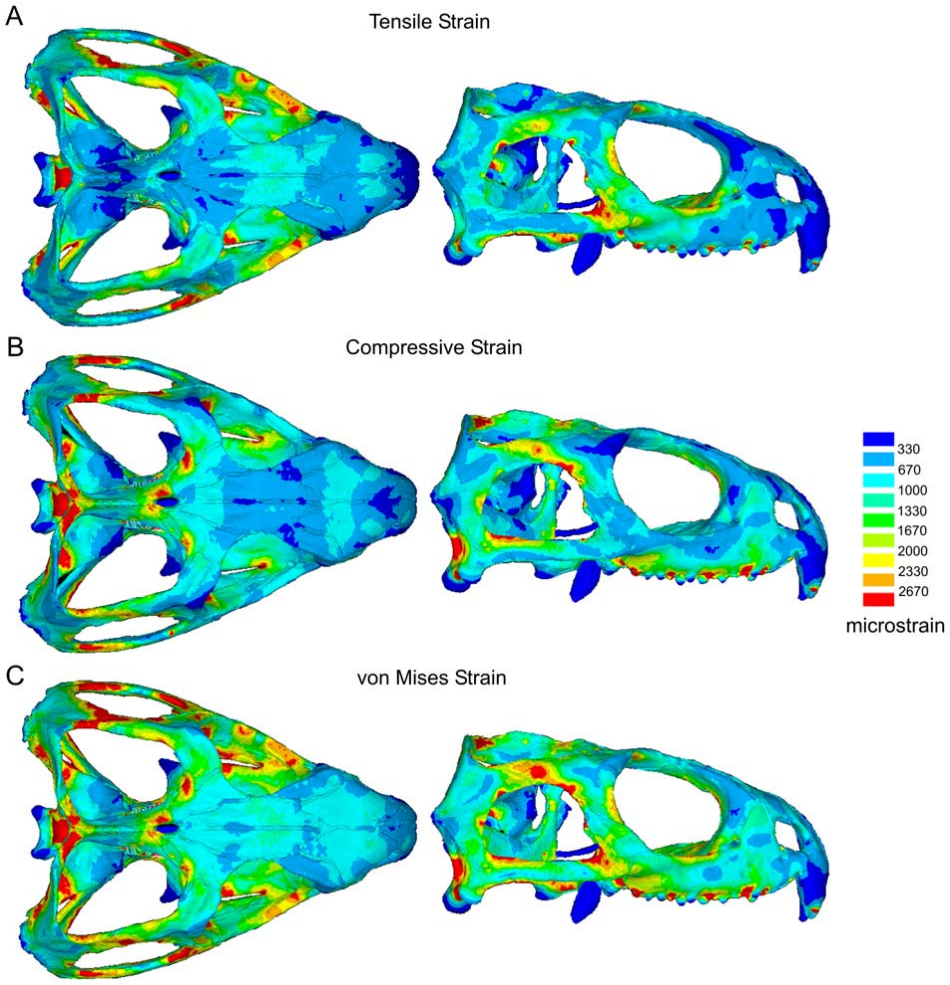
Combined loading tensile, compressive, and von Mises strain plots. Peak combined loading **A.** tensile, **B.** compressive, and **C.** von Mises strain plots.

When the individual peak element strains (i.e. the highest strain any one element ever experienced) are extracted from all fifteen individual biting analyses to generate a combined loading peak strain map, the obvious strain gradients (or heterogeneous strain magnitudes) noted during separate bites are considerably reduced (Figure 7). During combined loading 94.6%, 96.7%, and 98.0% of the skull experiences tensile, compressive, and von Mises strains of above 200 microstrain respectively when the peak element strains over all bites are considered (Figure 6). This compares to an average of approximately 70% during separate bites for all strain modes. Moreover, during combined loading 85.3%, 87.9%, and 91.1% of the skull in our model is at strains of between 400 and 2500 microstrain for tensile, compressive, and von Mises strain respectively, implying that the majority of the skull is shaped (remodelled) to keep strains within a specific tolerance range (Figure 6). Mean tensile, compressive, and von Mises strain over the entire skull (average strain across all individual finite elements in the model) is 784 microstrain, 887 microstrain, and 1140 microstrain when peak strains over all load cases are assessed. This value is typically only 500 microstrain during separate bites.

**Figure 8:**
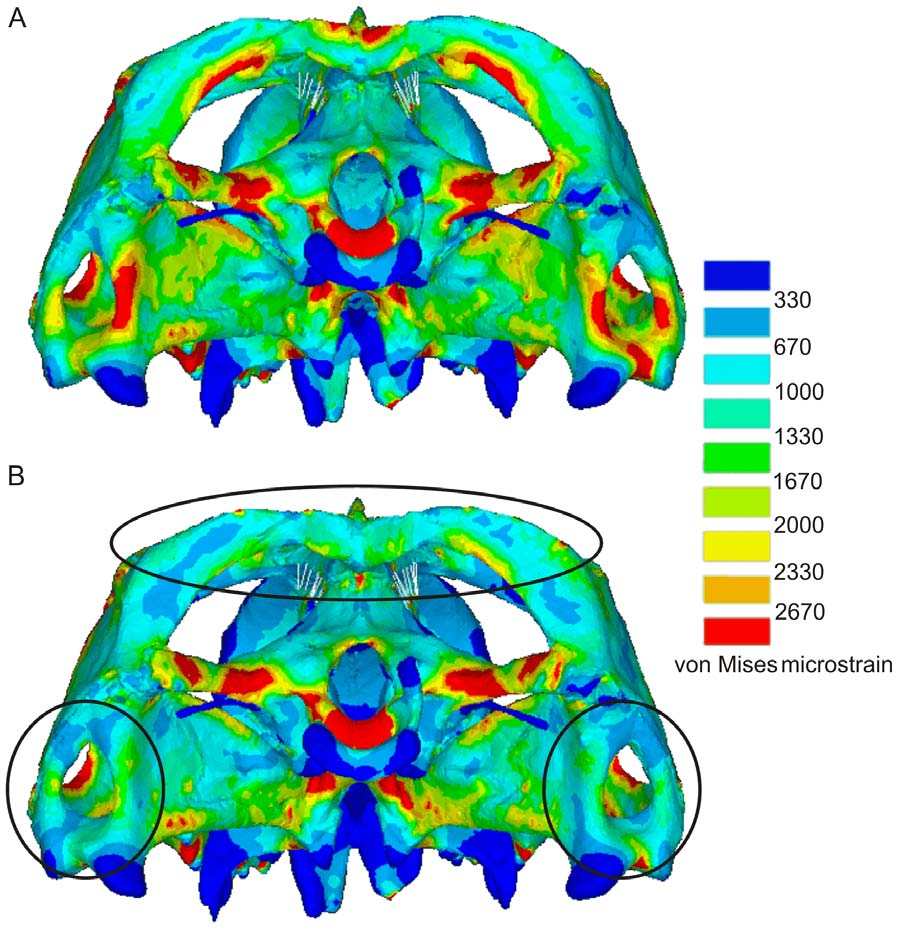
von Mises FEA plots with and without fascia forces. Posterior views of the skull showing von Mises strains predicted by the combined loading model. **A.** Without including fascial forces and **B.** including modelled fascial forces (see Figure 2A). Encircled regions highlight areas where strains have changed significantly due to the inclusion of the fascial forces.

Overall strain distributions over the skull remain largely unchanged with the addition of a fascial sheet over the upper temporal fenestra, but there were some striking reductions in localised peak strains, as highlighted in Figure 8. In particular, there is a reduction of peak strain on the lateral aspect of the postorbital bar where the jugal and postorbital meet, but the most obvious reductions in peak strains are on the posterior surface of the quadrate (encircled in Figure 8B), the temporal bar (squamosal and parietal, encircled in Figure 8B) and the posterior edges of the parietals where they meet in the midline (also encircled in Figure 8B). Localised peak strain areas around the perimeter of the upper fenestra were unaffected, with the exception of a small region on the posterior part of the postorbital.

## Discussion

The results of our comprehensive analysis implies that the form of the diapsid skull of *Sphenodon* is strongly linked to feeding forces. We show that both tensile and compressive peak strains are relatively evenly distributed throughout the skull when several loading cases are analysed (Figure 7). Although tensile strains are dominant in some regions of the skull, compressive strains are dominant in others (Figure 4). However, when analysing von Mises strain, which takes into account all principal strains, the distribution of strain is even more uniform when compared to tensile and compressive strains alone (Figure 7).

Our analyses show that over 91% of the skull is at von Mises strains of between 400 and 2500 microstrain when peak biting forces were analysed (Figure 6). While von Mises strain does not show which principal strain mode is dominant, making it difficult to interpret the exact response of the structure (e.g. whether or not it might fracture under tensile forces), von Mises strain does appear to be a good indicator of bone adaptation. *In vivo* studies predominantly on long bones have shown that both tensile and compressive strains are frequently experienced by bones during normal use, with peak strain during forceful loading ranging from 900 to 5200 microstrain [40]–[52]. In our analyses we find both high compressive and tensile strains over the skull, comparable in magnitude to those recorded experimentally in other animals (Figure 7), where compressive strains are dominant in approximately 60% of the skull (Figure 4). Focusing specifically on skulls, Herring et al. [53], [54] recorded strains of 2000–3000 microstrain when the masseter muscle was maximally contacted in a pig skull, peak values very similar to those predicted in our study.

Most literature on bone adaptation only refers to strain without inferring a particular mode, or even magnitude to this regard. What we do know is that bone adapts to mechanical loading, for example in experimental studies on adult rats, Robling et al. [55] showed bone to be deposited on both the tensile and compressive sides of artificially loaded forearms. Also under ‘normal’ loading situations, Haapassalo et al. [56] used peripheral quantitative computed tomography to show mean bilateral asymmetries (between the racket holding arm and non-racket holding arm) in second moments of area of the humeral midshaft in male tennis players. Although such studies show bone adaptation to functional loading, it is difficult to infer the exact strain magnitudes that initiate a particular bone remodelling effect. A figure published in Martin [57] does provide some suggestion into the approximate strain magnitudes that could cause bone adaptation. In this case, strains of below 50 microstrain are thought to represent disuse and thus bone resorption, whereas strains of between 1500 and 3000 cause some bone formation. Levels above 3000 microstrain are recognised as pathological overload and strains of between 50 and 1500 microstrain would generate equal bone resorption and formation rates (i.e. homeostasis). These values are only speculative and the strain mode or frequency is not specified, but our predicted von Mises strains in the skull of *Sphenodon* are comparable.

We simulated peak bite forces in our study (i.e. ∼140 N at an anterior bite position [36], [58]), and although bone needs to be able to withstand such forces without risk of failure, the majority of feeding forces will be significantly lower than these applied peak bite forces. For example, Aguirre et al. [59] showed that the approximate force needed to crush a beetle was 34 N, while Herrel et al. [60] recorded a value of 27 N to crush an egg. *Sphenodon* has a varied diet but it frequently includes beetles and occasionally sea bird eggs [61]–[63]. Thus, the force required to crush these foods is over four times lower than the peak bite force in *Sphenodon*. Scaling skull strains by a factor of four (i.e. in line with bite forces being four times lower) we show that over 91% of the skull is at strains of between 100 and 625 microstrain, well within the equilibrium window (i.e. equal bone resorption and deposition) as inferred by Martin [57].

The findings of this study imply that the skull of *Sphenodon* is adapted to feeding forces, with some regions adapted to tensile forces and others to compressive forces. Tendons and ligaments provide little resistance to compressive strains, and bone is necessary to provide compressive stability. We show that all regions of the skull experience compressive strain when all biting load cases are analysed, suggesting that it is mechanically necessary. However, while bone is necessary to resist compression, it must also be strong enough not to fail under tension. Therefore, once formed, bone must also adapt to tensile strains, and our results support this. Previous analyses, which include *in vivo* experimentation and FEAs suggest different functions for different regions of the skull based on stress and strain recordings/predictions [5], [64]–[69] (i.e. specific regions seem better suited to biting forces, bending strains, impact loads etc.). While our findings agree with this to some extent (e.g. a specific area of the skull may be linked to a specific bite point, or the forces generated at the jaw joints), they are not consistent with the conclusion that some regions of the skull are formed in relation to factors unrelated to functional strains (e.g. the idea that bone is formed to protect the brain and/or sensory organs from potential impact forces that have not yet occurred [5]). Previous studies did not take into account the full range of possible and potential loadings, a point made by Mikic and Carter [70] “one difficulty that is encountered when using bone strain data in studies of functional adaptation is the reported data are often far from a complete record of strain over an experimental period”. In relation to *in vivo* strain data, these authors further note that “reported results generally consist of a few average cyclic strain parameters that are extracted from a short period of recordings while an animal performs a very restricted task. Most investigators agree, however, that a much more complete record of strain history is required to relate bone biology and morphology to strain”.

In our study of skull function we found that strains resulting from a single bite do provide a limited view of overall skull performance (Figure 3 and Figure 6). When we considered a more complete range of physiological loads we showed strains to be more uniform over the entire skull (Figure 7). This finding suggests that the skull is well adapted to a range of functional strains. Although some regions appear to be adapted to tensile strains and others to compressive strains, all regions of the skull seem to be equally important with respect to overall feeding forces. We have shown that unilateral bites, in particular the more posterior unilateral bites, generate the highest strains across the skull. This suggests that such bites are more important to the morphology of the skull of *Sphenodon* than the bilateral ones.

The extent to which general skull form is determined by selection or growth remains uncertain, but our findings show that the skull of *Sphenodon* is optimally suited (mechanically ideal - or at least very well suited) to deal with the full range of loadings applied here. The term ‘optimally’ refers to the minimum amount of material (i.e. bone) necessary to ensure sufficient skull strength. An optimally formed skull as defined here will be more efficient than a sub-optimal, e.g. heavier skull form, in ensuring minimal bone volume, minimal weight, and also minimal energy demands in maintenance. For clarity, we would predict a non-optimised skull to display one of two contrasting conditions. It would either appear weak in relation to the normal forces applied to it, and experience very high and potentially damaging stresses and strains during normal loading, or, conversely, it might appear overly robust, with very low stresses and strains during normal loading and with excess bone mass that is not mechanically necessary. Since our findings infer that the skull of *Sphenodon* is well formed to resist the everyday forces applied to it, it is not unreasonable to suggest this may also be true for other diapsids with a frame-like skull.

Within our analyses a few small regions of high and low strain are present even when all fifteen biting load cases were accounted for. However, although the muscle representation is detailed in our models, some additional soft tissue structures, such as fascia and ligaments, were not included. At first consideration these structures may appear unimportant, but a recent study investigating the influence of the temporal fascia in primates has revealed that it might play a major role in the function of the skull [71]. Our analyses indicate that the fascial sheet stretched over the upper temporal fenestra in *Sphenodon* may also be significant (Figure 8). This fascial sheet is apparently tensed by upward bulging of the jaw adductor muscles (notably pseudotemporalis superficialis and adductor mandibulae externus medialis) as *Sphenodon* bites down on food (personal observations at Chester Zoo, UK; Dallas Zoo, USA). In this case the fascia serves to reduce peak strains (Figure 8), creating a more uniform strain distribution throughout the skull. The finding that the muscles (including the neck muscles), other soft tissue structures (e.g. upper temporal fascia), bite location, and joint forces all influence the strains within the skull suggests that modifications to any of these anatomical structures has the potential to affect skull form. This may even be somewhat applicable to the formation of unusual skull features, such as crests in chameleons, ceratopsians, and theropod dinosaurs [72]–[75].

The skull of *Sphenodon*, and probably other non-avian diapsid reptiles without a vaulted braincase (both extant and extinct), is adapted (in the sense of bone adaptation, rather than evolutionary development) to resist a range of load cases, not just single biting loads. The lower temporal bar, secondarily acquired in *Sphenodon* [66], [76]–[80] as well as in the common ancestor of archosaurs like crocodiles [66], [80], [81], is under compressive strain during all bites. This is consistent with previous suggestions that it provides a brace [66], [79], [82] that contributes to skull robusticity, and in large theropods such as *Tyrannosaurus rex* Osborn, 1905 and *Allosaurus fragilis* Marsh, 1877 this would be important as they would likely generate extremely large biting forces and experience heavy cranial loading [4], [83]. The corollary is that reptiles that lack a lower bar do not need a brace in this location. Early relatives of *Sphenodon* lack a lower temporal bar, the primitive condition for the group [76]–[79], but the dorsal position of the jaw joint in these small reptiles suggests that reaction forces would not have been directed along the lower temporal bar, had one existed [78], [84].

To conclude, our analysis of the skull of *Sphenodon* indicates that the bone has adapted to tensile and compressive strains generated during normal feeding activities. The combined peak von Mises strain distribution over the skull is relatively uniform, showing that all regions are strong enough mechanically to withstand normal everyday forces, while no region is overly robust and ‘over-designed’. Based purely on this finding, the skull form of *Sphenodon* can be considered optimal (mechanically ideal) in the sense that it comprises the minimal amount of bone material for the required skull strength. This optimal form is more efficient in terms of minimal bone volume, minimal weight, and minimal energy demands in maintenance over a sub-optimal, heavier skull form. While this study has not investigated potential forces associated with the brain, sense organs, and non-biting activities such as swallowing and tongue movements, its results are relevant to a broader understanding of skull form and not just to the skulls of diapsid reptiles. However, to test whether all skulls are optimally formed (sufficient strength with the minimal amount of material) with respect to bone strains (both tensile and compressive) would require the application of similar methods to other animal groups. Preliminary findings in macaques are encouraging in this regard (personal observations) but skulls with large vaulted braincases may be subject to additional quasi-static or high frequency low loads (e.g. associated with the brain) that could impact on skull form [28]–[30], [85].

## Materials and Methods

### MDA

Detailed descriptions of the MDA model development have been presented elsewhere [33], [36], [86]. Briefly, the skull and lower jaws (left and right parts) of a *Sphenodon* specimen (specimen LDUCZ x036; Grant Museum of Zoology, UCL, London, UK) were scanned in-house by micro-computed tomography (micro-CT), from which three-dimensional (3D) geometries were constructed using AMIRA image segmentation software (AMIRA 4.1, Mercury Computer Systems Inc., USA). Neck vertebral geometries were generated from additional micro-CT scans (specimen YPM 9194; Yale Peabody Museum of Natural History, New Haven, USA). These 3D geometries were imported into ADAMS multibody analysis software (version 2007 r1, MSC Software Corp., USA) in preparation for an MDA. Within ADAMS detailed muscle anatomy was incorporated onto the geometries, and accurate jaw joint and tooth contact surfaces were specified. Where the neck meets the skull a spherical joint was assigned that permitted the skull to rotate freely about all axes while constraining translational movements. The major adductor (jaw closing), depressor (jaw opening), and neck musculature were included, with each muscle group split into several sections and defined over the anatomical origin and insertions areas on the skull and lower jaws respectively [33], [86], [87] (Figure 2A). To permit biting, a food bolus was modelled that could be located at any position along the jaw, and a specially developed motion technique, named dynamic geometric optimisation (DGO), was utilised to open the jaw and to simulate peak biting. This motion technique, along with the muscle forces and biting performance, has been described and validated elsewhere [33], [36] (in reference to work carried out *in vivo* [58], [88]).

The biting simulations covered a range of biting types and locations, including four bilateral and eight unilateral bites at different tooth positions, a bite on the anterior-most chisel-like teeth, and two ripping bites that incorporate neck muscles (MDA model shown in Figure 2 and a summary of the simulations is shown in Table 1). During the ripping bites the jaws closed on a fixed food bolus, upon which and neck muscles were activated to lift (or try to lift) the head up and to the left, and up and to the right. These two ripping simulations ensured full activation of the neck muscles. During each simulation peak bite force, quadrate-articular joint forces, and muscle forces were predicted.

### FEA

The same 3D geometry constructed for the MDA skull was converted into a tetrahedral mesh consisting of 640,000 elements. The model was constructed from solid (ten node) higher order elements, which were specified with a Young's modulus of 17 GPa and a Poisson's ratio of 0.3 (consistent with direct measurements and within the ranges applied by others [1], [89]–[91]. Using the MDA predicted forces, a series of fifteen FEAs were carried out. Although theoretically all forces within the system should be in equilibrium, due to the large number of individual forces even small variations from the exact MDA locations of these applied forces causes instability within the FEAs (i.e. there would be unconstrained full body motion of the model). To ensure a stable FE solution, fixed constraints were included at the joint and bite contacts as defined by the MDA (i.e. neck joint, jaw joints, and bite point). One node at the neck location was constrained in the medial-lateral and anterior-posterior directions (x and z axes), one node at each jaw joint and bite point was constrained in the vertical direction (y axis).These constraints were considered minimal, and restricted rigid body motion but not deformations of the skull. For example, the neck, bite, and joint contact locations could all deform with respect to each other, and both jaw joint contact locations could deform relative to each other. After the FE solutions were complete, tensile (also known as maximum and 1^st^ principal), compressive (also known as minimum and 3^rd^ principal), and von Mises (also known as equivalent and mean) element strains of all 640,000 elements in the model were stored in element tables. In addition, the peak strain recorded in any one particular element during the fifteen separate simulations was extracted and combined to map the peak strains across the skull. This is referred to as a combined loading model.

An additional investigation was carried out to understand the influence of other *non-bone* structures. To this end we simulated an upper temporal fascial sheet, which is likely tensioned by large superior bulging of the jaw adductor muscles during biting (personal observations from animals at Chester Zoo, UK; Dallas Zoo, USA). Here we applied a total force of 133 N around the perimeter of each upper temporal fenestra (7 N over 19 force vectors – see Figure 2A). This magnitude was based on an unrelated investigation [71], where the total fascial force was found to be approximately 85% of the muscle force applied by an associated muscle group(s). In this case the associated muscles were pseudotemporalis superficialis and adductor mandibulae externus medialis [36], [87].
